# Narratives to enhance smoking cessation interventions among African-American smokers, the ACCE project

**DOI:** 10.1186/s13104-015-1513-1

**Published:** 2015-10-14

**Authors:** Andrea Cherrington, Jessica H. Williams, Pamela Payne Foster, Heather L. Coley, Connie Kohler, Jeroan J. Allison, Catarina I. Kiefe, Julie E. Volkman, Thomas K. Houston

**Affiliations:** Division of Preventive Medicine, University of Alabama at Birmingham, Birmingham, AL USA; Department of General Internal Medicine, University of Alabama at Birmingham, Birmingham, AL USA; Rural Health Institute for Clinical and Translational Science, University of Alabama School of Medicine, Tuscaloosa Campus, Tuscaloosa, AL USA; Department of Community and Rural Medicine, University of Alabama School of Medicine, Tuscaloosa Campus, Tuscaloosa, AL USA; Department of Health Behavior, University of Alabama at Birmingham, Birmingham, AL USA; Quantitative Health Sciences and Medicine, University of Massachusetts Medical School, Worcester, MA USA; Center for Health Quality, Outcomes and Economic Research (CHQOER), Bedford, MA USA; e-Health Quality Enhancement Research Initiative (QUERI) Center, Bedford VAMC, Bedford, MA USA; Center for Healthcare Organization and Implementation Research (CHOIR), Bedford, MA USA; Department of Veterans Affairs, ENRM Veterans Hospital, 200 Springs Rd., Bedford, MA 01730 USA

**Keywords:** Smoking cessation, Narratives, African-American

## Abstract

**Background:**

Low-income, African-American smokers are less likely to have resources to aid in quitting smoking. Narrative communication may provide an enhancement to traditional smoking cessation interventions like NRT, medications, or behavioral treatments for this audience. After extensive pilot testing of stories and personal experiences with smoking cessation from African-Americans from a low-income community, we conducted a randomized control trial using stories to augment routine inpatient treatment among African-Americans at an urban Southern hospital (N = 300).

**Results:**

Differences in smoking cessation outcomes between the intervention (stories DVD + routine clinical treatment) and control (routine clinical treatment) arms were compared using self-report and carbon monoxide measurement at 6-months. Compared to control, individuals who viewed the intervention stories DVD reported greater intentions to quit. Although continuous quitting marginally favored the intervention, our main result did not reach statistical significance (p = 0.16).

**Conclusion:**

Narrative communication via storytelling to promote smoking cessation among African-Americans in the South is one method to communicate smoking cessation. Results suggest this may not be sufficient as a stand-alone augmentation of routine clinical treatment for continuous smoking cessation. Smoking cessation efforts need to continually assess different means of communicating to smokers about quitting.

Clinical Trials Registration: The ClinicalTrials.gov Identifier is NCT00101491. This trial was registered January 10, 2005

## Background

Smoking rates among African-Americans remains a significant concern for public health, as African-Americans are at an increased risk for lung cancer [1]. Although more than 70 % of African-American adult smokers indicate that they want to quit smoking, prevalence of cessation is still higher among Whites than among African-Americans [2]. In addition, compared to Whites, African-American smokers are less likely to report having been told by their doctor to quit smoking and less likely to have resources to aid in quitting [3]. Furthermore, smoking rates increase for African-Americans at lower educational levels [1] and rates are higher in the South (21 % in 2010) compared to other regions of the United States [4]. It is important to understand interventions that can motivate African-Americans to stop smoking to reduce these disparities.

An increasing body of literature suggests narrative communication, or storytelling, may be an effective means to promote behavior change in African-American communities [5–8] and sharing of peer experiences can be effective for smoking cessation [9]. Storytelling is a critical part of the African-American oral tradition and culture [10], and the difficulties associated with smoking cessation among African-Americans [11], lend to the potential to use narratives as a way to enhance traditional smoking cessation interventions. This study represents a randomized control trial of personal stories and narratives to increase smoking cessation behaviors previously piloted [12] among African-Americans and offers considerations for the effectiveness of narratives for cessation interventions.

### Narrative communication for behavior change

The persuasiveness of narrative communication lies in the extent to which it can engage the viewer through realism and homophily, or similarity between that which is portrayed and the viewer [13, 14]. The underlying assumption is hearing a person’s story or experience is considered credible and trustworthy, therefore can potentially motivate and persuade individuals towards behavior change [15]. By sharing stories that feature someone to whom the viewer can relate, it reduces counter-arguing and resistance to the message [15, 16]. Stories can add meaning to health issues [5] and help viewers visualize themselves engaging in actions taking place [12].

The use of narratives in traditional health intervention is being used frequently in health promotion and interventions for African-Americans. In a previous randomized trial, Houston et al. [12] found DVDs of African-Americans sharing their stories managing hypertension resulted in significant improvements in blood pressure for patients with baseline uncontrolled hypertension. Further, rural African-American women exposed to stories of hope from African-American cancer survivors increased their use of breast self-examination and mammography [7]. In a separate, smaller study, African-Americans with diabetes who received a culturally sensitive intervention including storytelling showed a trend towards improvements in self-care, goal attainment, and empowerment [6]. Thus, narrative communication provides a way to discuss health behavior changes among African-Americans. However, we found no published research trials using storytelling for smoking cessation.

### Modeling of behavior for smoking cessation via peer experiences

As smoking cessation can be a hard and arduous process, hearing from African-Americans with similar experiences and struggles with smoking cessation may be beneficial to other African-American smokers towards behavior change. Narrative communication can provide the personal and peer experience that is valuable in helping others learn and make life decisions [5]. Recently, the CDC is using peer experiences on smoking as part of a mass media campaign to increase awareness of government smoking cessation resources; preliminary results show the effort and focus on peer experiences to be effective [9].

An added persuasive element of narrative communication is the ability to show others, or model, experiences associated with a behavior. Following the tenets of Social Cognitive Theory (SCT) [17] that individual behavior is inherently situated in social interactions and that observational behavior can lead to behavior change, narrative communication lends itself to enabling modeling and observational learning to occur through the telling of personal experiences. Given the difficulty in smoking cessation for many African-Americans [11], SCT can help identify key concepts that can contribute to the modeling of smoking cessation behaviors. In particular, self-efficacy and outcome expectancies are important towards intentions to quit smoking. Viewing stories by fellow African-Americans can offer others a way to enhance expectations that they are capable of quitting, or increase perceptions of self-efficacy as well as increasing expectations that a positive outcome will occur such as maintaining a quit rate (outcome expectancies).

In addition, peer experiences can address culturally relevant experiences for African-Americans [18]. In 2008, clinical guidelines on smoking cessation interventions included a recommendation for increased attention to augmenting routine clinical care with the development and evaluation of smoking cessation interventions for racial and ethnic minorities [19]. A key aspect of these interventions focusing on racial and ethnic populations is ensuring the cultural relevance of their studies for the population. Narratives by peers that have successfully quit smoking are one way to address cultural norms and sensitivities that may be important challenges and barriers to smoking cessation. For instance, a culturally tailored DVD intervention reported higher rates of smoking cessation for those who both viewed the tailored intervention and identified with the race of the storyteller [20]. Taken together, we hypothesize:

1. African-American smokers receiving the Storytelling DVD intervention in addition to routine clinical treatment will be more likely to report positive intentions to quit compared to smokers in a control group receiving routine clinical treatment and an attention control DVD.
2. African-American smokers receiving the Storytelling DVD intervention will be more likely to quit smoking at 2-week and 6-month post-intervention, compared to those patients in the control DVD group at 2-week and 6 month follow-up.
  To present an ideal time to present a smoking cessation intervention, we focused on patients with chronic illness at an urban Southern hospital. Individuals hospitalized may feel more susceptible and vulnerable to negative outcomes associated with smoking, and therefore more open to smoking cessation efforts [21]. We therefore hypothesize:
3. Success of the smoking cessation intervention will be modified by self-reported health status in the hospital.

## Methods

### Study design

A randomized controlled trial was conducted to test the impact of an interactive storytelling DVD on processes of change and 6-month cessation rates for these low-income, low-literacy, African-American smokers with chronic illnesses. The DVD was provided as an augmentation of standard medical care. As the majority of these smokers were not preparing to quit or actively quitting, this Storytelling DVD trial can be considered a cessation induction intervention designed to motivate patients to quit, regardless of their readiness to quit.

### Participants

Medical teams from the inpatient wards at an urban, safety net hospital in the Southern U.S. referred African-American smokers to the research team from April 2006 to December 2008. The patients were approached by research assistants to verify eligibility: age 19 and older, self-identified African-American race/ethnicity, current smoker at the time of admission, and absence of hearing, vision, or comprehension difficulties. Patients were excluded if they had a primary diagnosis of alcoholism, drug dependency, serious mental illness (schizophrenia or bipolar), were incarcerated, or were unable to participate in a phone call following discharge. The patients were active smokers, admitted to the hospital with a number of chronic conditions (see Table 1).

**Table 1:** Participant characteristics in the stories randomized trial

	Intervention group	Control group	*P*
	*n* (%)	*n* (%)	
Age (years), mean ± SD	50.4 ± 10.0	49.4 ± 10.3	0.42
Gender, female	83/150 (55.3)	74/150 (49.3)	0.30
Educational level			
Elementary (Grades 1–8)	5/150 (3.3)	3/150 (2.0)	0.92
Some high school (Grades 9–11)	34/150 (22.7)	34/150 (22.7)	
High school graduate (Grade 12 or GED)	62/150 (41.3)	62/150 (41.3)	
Some college/Tech. school (1–3 years)	40/150 (26.7)	39/150 (26.0)	
College graduate (≥4 years)	9/150 (6.0)	12/150 (8.0)	
Self-assessed income for basic needs (food, housing, clothing, medical care) adequate at baseline			
Yes	36/139 (25.9)	29/136 (21.3)	0.37
No	103/139 (74.1)	107/136 (78.7)	
Health status self-assessment			
Excellent or very good	14/150 (9.3)	15/150 (10.0)	0.98
Good	35/150 (23.3)	36/150 (24.0)	
Fair	60/150 (40.0)	63/150 (42.0)	
Poor	23/150 (15.3)	21/150 (14.0)	
Don’t know/not sure	18/150 (12.0)	15/150 (10.0)	
Admitting diagnosis			
Cardiovascular/stroke	57/146 (39.0)	54/146 (37.0)	0.93
Pneumonia/asthma/other pulmonary	20/146 (13.7)	17/146 (11.6)	
Diabetes	10/146 (6.9)	8/146 (5.5)	
Cancer	11/146 (7.5)	12/146 (8.2)	
Renal	9/146 (6.2)	8/146 (5.5)	
Other	39/146 (26.7)	47/146 (32.2)	
Readiness to quit smoking			0.4
1	107/145	110/143	
2	33/145	25/143	
3	5/145	8/143	
Number of cigarettes smoked per day			
<10	66/144 (45.8)	86/146 (58.9)	0.009
11–20	56/144 (38.9)	53/146 (36.3)	
21–30	15/144 (10.4)	3/146 (2.1)	
>31	7/144 (4.9)	4/146 (2.7)	

African American inpatients in the southern U.S, April 2006 to December 2008

### Procedure

After approaching potential participants and verifying eligibility, research assistants obtained written informed consent. After providing consent, participants: (a) completed a baseline survey; (b) received tobacco cessation information via brief counseling based on their responses to readiness to quit; (c) were provided a DVD player to use in the hospital room and to take home to assure access to the DVD after discharge; and (d) viewed either the intervention DVD or control DVD as determined by block randomization. The face-to-face baseline survey determined smoking habits as well as general health status and a pre-behavioral self-reported assessment.

### Randomization

For randomization we used blocks of 10 to balance allocation. Randomization was concealed for the patient as all participants received an interactive DVD. Only half received the one with Stories. We did not reveal that the goal of the intervention was to study storytelling, so all participants received an “intervention.” Further, the DVDs (intervention and control) were ordered in the order of the randomization table, and the intervention or control DVD placed inside and sealed. Thus, randomization status was concealed for the research staff until the seal on the DVD was broken. The staff conducting follow-up calls was different than recruiting staff, and these staff were blinded to intervention or control status.

The DVD intervention was provided as an *augmentation* of standard medical care in the hospital (and follow-up outpatient care), including smoking cessation counseling and treatments including nicotine replacement therapy. The inpatient clinical providers and teams were responsible for choosing nicotine-replacement therapy and other pharmaceutical treatments, and could also provide brief counseling and patient education materials. We monitored the care received by patients using chart review.

### Data collection

The research assistant administering the baseline hospital survey was blinded to intervention or control status until the DVD was opened. After patients viewed the intervention or control DVD, a follow-up survey was immediately conducted to assess participants’ level of engagement with the video, as well as the influence of the video on intentions to change smoking behavior. For completing the hospital interview, patients received a DVD player and a copy of the intervention or control DVD. Participants were not required to view the entire DVD, but were asked to explore the interactive menus for as long as they wished. The protocol was approved by the University of Alabama at Birmingham’s Institutional Review Board and the review board of Cooper Green Mercy Hospital. The entire study procedure took approximately 60 min.

Patients were followed-up by phone at 2 weeks and 6 months after the baseline hospital interview to assess self-reported smoking status. For each survey, patients received a $15 gift card. After the 6-month telephone interview, patients who reported quitting were asked to confirm cessation by carbon monoxide validation at the hospital for which they received a $15 gift card for their time and transportation costs. Additional patient data was abstracted from the medical record by blinded, trained abstractors with a structured medical record review instrument using the publicly available MedQuest software.

### The intervention DVD

A DVD-delivered intervention was developed that included current and former smokers discussing their stories of smoking cessation. Participants featured in the DVD were recruited from the same urban, safety net hospital that was used for the subsequent randomized trial. Details of the development protocol have been published [12]. The four storytellers, or stories, included in the DVD represented clear messages linked to concepts from SCT, including direct verbal persuasion, outcome expectations, outcome expectancies, and direct experience. After each 5-min story, a “Learn More” didactic segment harkened back to the story and emphasized the key persuasive content. The “Learn More” content branched into ready to quit and thinking of quitting content to maintain smoking cessation guideline-concordant information and designed to augment the information highlighted in the stories (e.g., talking to your doctor, family, motivation, using patches, identifying reasons to quit) [12]. Following the stories and “Learn More” segments, viewers could choose between two further educational videos targeted to their readiness to quit smoking (one entitled “Thinking about Quitting” and one “Ready to Quit Plan”).

### The control DVD

An attention-control DVD was developed that included five brief health-related mini-lectures (e.g., non-culturally tailored, non-narrative, and non-tobacco-related).

### Measures

Immediately after viewing the DVD, smokers’ level of engagement with the DVD was measured using a Video Transportation Scale [22], a modification of the original Green and Brock [15] scale (5 of items). To measure the delivery and dose of the intervention, participants were asked to report how many stories, “Learn More” segments, and quit plans they had viewed. In addition, participants were asked to identify which of the smoker stories they viewed.

Readiness to quit was asked as a single item with four responses assessing whether participant planned to quit in the next 30 days, 6 months, did not plan to quit, or had already quit. Data from the medical record allowed us to determine admitting diagnoses and standard of care treatments (including pharmaceuticals and counseling).

From Social Cognitive Theory, we assessed behavioral intentions to quit (“Hypothesis 1”). Intentions to quit smoking were measured along seven items on a 100-point scale asking how much the video influenced you to change key smoking related behaviors (e.g., cut down on smoking or set a quit date). This scale (Table 2) was adapted from a validated scale used by Kohler et al. [23].

**Table 2:** Behavioral intentions to quit smoking in the intervention and control group after randomization and watching the DVD

	Intervention group	Control group	*P*
	N = 150	N = 150	
	Mean (SD)	Mean (SD)	
I am motivated to:			
(a) Cut down on smoking	75.6 (26.3)	62.5 (33.3)	<0.01
(b) Quit smoking	84.9 (23.1)	65.8 (35.3)	<0.01
(c) Talk to a doctor about quitting smoking	75.8 (32.5)	64.0 (35.7)	<0.01
(d) Get support from those around you to help quit smoking	77.9 (30.1)	63.6 (35.6)	<0.01
(e) Set a quit date	73.0 (33.2)	56.6 (36.8)	<0.01
(f) Use nicotine replacement therapy like the patch or gum	67.8 (36.6)	51.0 (41.7)	<0.01
(g) Make list of reasons to quit smoking	77.1 (32.7)	65.9 (34.8)	<0.01

Our main outcome (“Hypothesis 2”) was 2-week and 6-month follow-up of tobacco use was also assessed. Two-week quit was defined as not smoking even one cigarette in the last 7 days. Six-month follow-up was assessed as 7 day point prevalent smoking cessation. Of the participants reporting smoking cessation at 6 months, we measured carbon monoxide levels to verify cessation.

### Data analysis

To characterize the study sample and assess the success of randomization, the intervention and control group characteristics were compared using Chi square and two-tailed t-tests as appropriate for categorical and continuous variables.

### Hypothesis 1

To evaluate the impact of the DVD on intention to change smoking-related behaviors, the difference in post-intervention behavioral constructs (behavioral intentions, self-efficacy, outcome expectancy, and readiness to quit) were assessed by comparing the proportion responding positively to each individual measure for intervention and control using Chi square and two-tailed t-tests, as appropriate.

### Hypothesis 2

To test our hypothesis 2 smoking cessation outcomes, differences in smoking cessation outcomes by intervention and control was compared using Chi square tests. First, differences in sustained cessation 2 weeks after discharge was compared, then, 6-month continuous self-reported cessation was assessed, and cessation confirmed by carbon monoxide. In the primary intent-to-treat analyses, all those lost to follow-up in the intervention and control group were considered still smoking, as discussed in the Society for Research on Nicotine and Tobacco recommendations and recent drug and behavioral intervention studies [24]. This study was powered to detect a difference in smoking cessation between 13 and 14 %, with power = 0.8, and alpha 0.05, assuming a control group rate of 15–16 % following recommendations from a 2008 Cochrane Review [25].

In addition to the primary intent-to-treat analysis, a multivariable logistic regression analysis adjusting for important predictors of 6-month cessation was conducted. Again in this analysis, the dependent variable was 6-month continuous cessation (assigning those lost to follow-up as smoking) and the main independent variable was randomization group (see Table 3). Additional analysis was conducted using multiple imputation of missing follow-up data. Supporting the intent-to-treat analysis, and as recommended by the Society for Research on Nicotine and Tobacco [24], a per protocol analysis was conducted including only those with 6-month follow-up data again comparing intervention and control using Chi square.

**Table 3:** Two-week and 6-month smoking cessation outcomes comparing stories intervention and control

	Intervention group, *n/N* (%)	Control group, *n/N* (%)	*P*
Self-reported quit^a^ at 2 weeks	65/150 (43.3)	55/150 (36.7)	0.24
Self-reported continuous quit at 6 months	50/150 (33.3)	39/150 (26.0)	0.16
Continued quit at 6 months, carbon-monoxide verified	23/150 (15.3)	17/150 (11.3)	0.31

^a^Quit is defined as not smoking even one cigarette in the last 7 days

### Hypothesis 3

Finally, we also tested the hypothesis that there would be effect modification by self-reported health. To evaluate this effect, stratified analyses by self-reported health status (fair/poor versus good/excellent) was conducted using Chi square to assess the difference in 6-month continuous cessation (assigning those lost to follow-up as smoking) by randomization group. All analyses were conducted using the STATA 10.0 statistical package in 2010.

## Results

### Patient characteristics and baseline readiness to quit

**Fig. 1:**
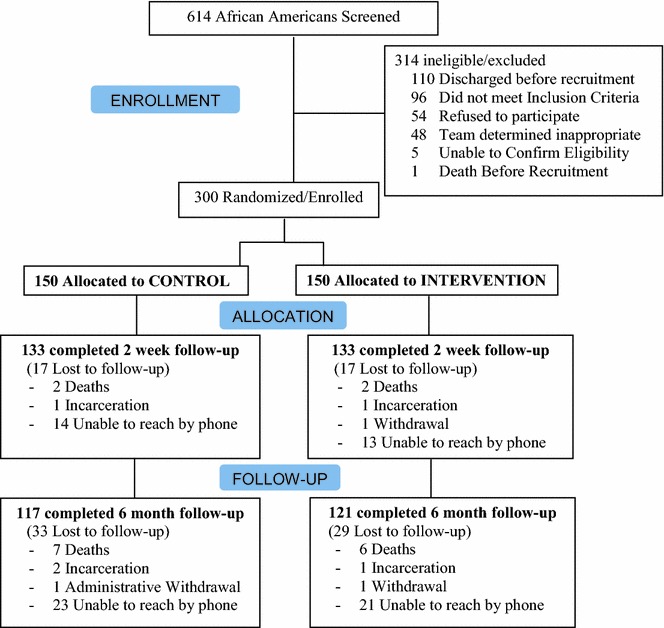
Stories consort diagram

Three hundred African-American smokers from the inpatient service of the safety-net hospital were recruited and randomized. Participation rates were high (see CONSORT diagram, Fig. 1). African-American smokers had a mean age of 50 (*SD* = 10) and 52 % were female. Few (7 %) had some college education, and 25 % had less than a high-school education. Overall, 79 % reported that their income was not adequate to meet their basic needs. Patients presented with a variety of medical illnesses (Table 1), and most reported fair or poor health status.

As Table 1 shows, few differences were noted between the two randomized groups, although intervention patients more frequently smoked over 20 cigarettes per day. Baseline readiness to quit was similar across the two groups.

### Standard care delivered by the clinical teams

All (100 %) received initial brief counseling from the inpatient nursing staff, as required by the hospital. Smoking Cessation patient education materials were provided to 82 % (n = 123) of participants in the control condition, and 86.67 % (n = 130) in the intervention condition. In addition, 54 % (n = 81) in the control condition had chart documentation that they received additional smoking counseling by their physician, with 56.67 % (n = 85) in the intervention condition having chart documentation of additional counseling by their physician (p = 0.6). In both the intervention and control groups, approximately 3 % (N = 5 intervention, N = 4 control) received inpatient nicotine replacement therapy (p = 0.7).

### Viewing the DVD inpatient (intervention fidelity)

All intervention participants reviewed at least part of the DVD. Forty-six percent (69/150) of intervention participants went on to view at least one “Learn More” segment after watching a story. Only 30 % of participants went on to view the “Ready to Quit” Plan (32/150) or “Thinking about Quitting” section (15/150).

### Impact of storytelling DVD on behavioral intentions (H1)

As demonstrated in Table 2, immediately after watching the DVD, patients in the intervention group were more likely to positively respond to all seven behavioral intentions, including the patient reports of motivation to cut down on smoking, quit smoking, talk to a doctor about quitting, get support from those around you, set a quit date, use nicotine replacement therapy, and make a list of reasons to quit smoking.

However, at 2-week follow-up, there were no differences in self-reported readiness to quit, quitting self-efficacy and outcome expectancy between the intervention and control groups.

### Impact of storytelling DVD on 2-week and 6-month smoking cessation (H2-primary outcome)

Overall, 79 % of participants completed 6-month follow-up and there was no difference in attrition by randomized group. In the intent-to-treat analysis, when assuming those lost to follow-up had relapsed, overall, 60 % (180/300) of these hospitalized African-American smokers had relapsed to smoking by 2 weeks. Two-week point prevalence cessation was higher in intervention (43.3 %) than control (36.7 %), although not statistically significant (see Table 4).

**Table 4:** Multivariable logistic regression model predicting cessation and 2 weeks and 6 months

	Self-reported quit at 2 weeks	Self-reported continuous quit at 6 months	Continued quit at 6 months, carbon-monoxide verified
Randomization group			
Storytelling DVD intervention	1.03 (0.59–1.80)	1.39 (0.75–2.56)	1.21 (0.56–2.62)
Control	1.0	1.0	1.0
Readiness to quit			
Ready to quit smoking	6.72 (3.0–15.1)	12.20 (3.62–41.11)	3.83 (1.11–13.15)
Not ready to quit	1.0	1.0	1.0
Self-reported health status			
Fair/poor health	1.30 (0.73–2.33)	1.21 (0.64–2.29)	1.08 (0.48–2.42)
Good/very good/excellent health	1.0	1.0	1.0
Cigarettes per day			
1	1.0	1.0	1.0
2	0.77 (0.43–1.39)	0.57 (0.30–1.10)	0.90 (0.40–2.04)
3	0.60 (0.18–2.02)	0.36 (0.09–1.48)	0.35 (0.04–3.02)
4	0.80 (0.17–3.78)	0.72 (0.12–4.37)	0.75 (0.08–7.18)
Gender			
Female	1.13 (0.65–1.98)	1.72 (0.92–3.22)	1.05 (0.48–2.29)
Male	1.0	1.0	1.0
Age (years)	1.04 (1.01–1.07)	1.05 (1.01–1.08)	1.05 (1.01–1.09)

**Table 5:** The teachable moment: 6-month cessation stratified by self-reported health status

	Intervention group, *n/N* (%)	Control group, *n/N* (%)	P
Good-to-excellent health status			
Self-reported quit at 2 weeks	17/49 (34.7)	21/51 (41.2)	0.50
Self-reported continuous quit at 6 months	11/49 (22.5)	16/51 (31.4)	0.32
Fair/poor health status			
Self-reported quit at 2 weeks	37/83 (44.6)	30/84 (35.7)	0.24
Self-reported continuous quit at 6 months	30/83 (36.1)	19/84 (22.6)	0.055

For 6-month cessation, both self-reported continuous quitting and continuous quitting with 6-month carbon monoxide validation was assessed. Of those reporting cessation, 62 % were available for in-person CO validation. There were no statistically significance differences between groups for either measure (*p* = 0.16; Table 4) and this remained the case after adjustment (Table 3) for readiness to quit, self-reported health status, cigarettes per day, gender and age.

### Per protocol analysis and effect modification by self-reported health status

The results were further analyzed including only those completing 6-month follow-up (N = 238) in a per protocol analysis. Though intervention participants were more likely to quit compared with control, the difference was not statistically significant (41 vs. 33 %, *p* = 0.2). Among those with self-reported good or excellent health, 6-month self-reported continuous cessation was low (23 %), and did not favor the intervention (Table 5). However, among those with self-reported fair or poor health, 6-month self-reported continuous cessation favored the intervention and did not reach significance at p = 0.055 (36.1 % intervention versus 22.6 % control).

## Discussion

This study utilized a randomized controlled trial to assess the effect of a narrative communication intervention on smoking cessation as measured by self-report as well as biochemical verification. While the intervention did not increase quit rates at 6-months, results do suggest that patient narratives delivered via interactive DVD combined with current cessation treatment in a hospital setting may be an effective means to move the needle on intention to quit among hospitalized African-American smokers.

Immediately after watching the storytelling or control DVD, participants were asked a set of seven behavioral intentions that we predicted might be motivated to perform, and would be influenced by the DVD. The questions allow us to understand the proximal internal behavioral motivations of the patients, and understand the comparative impact the DVD may have had on these motivated processes. Uniformly, patients expressed higher endorsement of all seven intentions immediately after watching the DVD. Thus, looking into the “black box” and trying to understand how the DVD is working, we find that engagement in the stories increased initial motivation, and intention to quit was sustained at 2 weeks.

Compared to participants in the control group, participants in the intervention group had increased intention to quit smoking and readiness to quit at 2 weeks. In this study, the brief intervention was designed to come from African-American community members in the form of a story. Several studies provide evidence for narrative communication as a mechanism of behavior change, ranging from prevention and cancer screening to chronic disease management [5]. Taken together, the results from the current study and others suggest storytelling can produce change in theory-based constructs linked to behavior change, although difference in 6 month cessation was not found.

Results from this study also support and advance previous literature suggesting that capitalizing on a teachable moment, in this case hospitalized smokers who perceive themselves in poor health, may enhance the likelihood of success in a smoking cessation intervention. The phrase “teachable moment” has been used to describe “naturally occurring health events thought to motivate individuals to spontaneously adopt risk-reducing behaviors” [26]. A recent Cochrane review demonstrated that when smokers are hospitalized, for example, intensive counseling plus 1 month follow-up increased the odds of smoking cessation by 65 % 6–12 months after hospital discharge [27]. Interestingly, these findings held up regardless of the reason for hospital admission. While hospitalization may generally represent a teachable moment for smoking cessation, the results from the current study nevertheless suggest that assessing how a patient self-rates their own health status may help identify those for whom the teachable moment may be particularly salient.

All studies have limitations. As we report, we had some loss to follow-up, and were not able to carbon monoxide verify every smoker. There is the possibility that participants did not relate to the storyline or the characters presented on the DVD which may then have affected quit rates. Because both intervention and control smokers received high levels of brief advice and educational materials, the standard care may have been stronger that the relative impact of the DVD intervention. Also, our study was only powered to detect a difference of 13 % of 6-month cessation. A larger sample size would have had more power to significantly detect the four percent difference in carbon monoxide verified cessation in this study.

## Conclusion

This intervention used narrative communication and storytelling to create a smoking cessation intervention for hospitalized, low-income African-Americans living in an urban setting in the South. While the results suggest this approach may be insufficient as a stand-alone intervention, it may, nevertheless, be a step in the right direction by increasing intention to quit, particularly among those who perceive themselves to be in poor health. While the intervention was specialized to a specific population, this did allow us to develop a locally-targeted intervention to enhance potential effectiveness for a specific community. It was designed to be brief, and interactive to increase viewers’ engagement and to avoid being overly didactic. However, brief interventions aimed at smoking cessation tend to require some follow-up counseling or support in order to effect change [28]. Future studies should explore whether a brief storytelling intervention for hospitalized smokers could be augmented by subsequent outpatient behavioral counseling and support to improve smoking cessation rates long-term. In this study, the intervention’s brevity and low intensity may have been a significant limitation. Also, the use of carbon monoxide to indicate sustained abstinence and the limited self-report measures may have contributed to the non-significant findings.

### Practice implications

Results from this study offer further evidence towards smoking cessation programs and augmenting current practices with additional communication means. Storytelling, and specifically the sharing of peer-experiences, highlights an additional avenue to pursue for smoking cessation program. The difficulties associated with smoking cessation, and maintaining a quit rates, indicate that peer experiences may benefit others in long term success. Smoking cessation programs may want to investigate the use of peer experiences in future endeavors.

## Abbreviation

SCT: social cognitive theory
